# Prognostic value of Epstein-Barr virus DNA load in nasopharyngeal carcinoma: a meta-analysis

**DOI:** 10.11604/pamj.2022.41.6.28946

**Published:** 2022-01-03

**Authors:** Imane EL Alami, Amina Gihbid, Hicham Charoute, Wafaa Khaali, Selma Mohamed Brahim, Nezha Tawfiq, Rachida Cadi, Khalid Belghmi, Mohammed El Mzibri, Meriem Khyatti

**Affiliations:** 1 Laboratory of Viral Oncology, Institut Pasteur du Maroc, Casablanca, Morocco,; 2 Laboratory of Microbiology, Pharmacology, Biotechnology and Environment, Faculty of Sciences Ain Chock, Hassan II University, Casablanca, Morocco,; 3 Laboratory of Pathophysiology, Molecular Genetics and Biotechnology, Faculty of Sciences Ain Chock, Hassan II University, Casablanca, Morocco,; 4 Mohammed VI Center for Cancer Treatment, Ibn Rochd University Hospital, Casablanca, Morocco,; 5 Biology and Medical Research Unit, National Center of Energy, Nuclear Sciences and Techniques Rabat, Morocco

**Keywords:** Epstein-Barr virus, nasopharyngeal carcinoma, Epstein Barr Virus DNA load, prognostic

## Abstract

The present meta-analysis was conducted to evaluate the prognostic value of pre and post-Epstein Barr Virus (EBV) DNA load testing and to assess the clinical benefit of using this molecular approach in the prognosis for a better nasopharyngeal carcinoma (NPC) management. Relevant studies were searched in different database until May 2020. Patient´s outcomes overall survival (OS), disease free survival (DFS), progression-free survival (PFS), distant-metastasis-free survival (DMFS), and local-regional-failure-free survival (LRFS), hazard ratios (HRs) and 95% confidence intervals (CIs) were extracted from selected studies. The association of pre and post-EBV DNA load and survival outcomes was assessed using review manager and the pooled HRs with 95% CIs were calculated. Twenty-six eligible studies were included in this meta-analysis, with a total of 9966 patients. Pooled HRs showed that EBV DNA levels before and after treatment are significantly associated with survival outcomes, with HR (95% CI) of 2.09 [1.74, 2.51] for OS, 1.77 [1.19, 2.62] for DFS, 2.53 [2.18, 2.92] for DMFS, 1.78 [1.45, 2.19] for LRFS and 2.17 [1.91, 2.47] for PFS in pre-EBV DNA, and an HR (95%) of 4.52 [2.44, 8.36], 4.08 [2.38, 6.99], 5.59 [ 3.58, 8.71] and 8.88 [5.29, 14.90] for OS, DFS and PFS and DMFS in post-EBV DNA, respectively. High pre and post-EBV DNA levels were significantly associated with poor NPC patient´s survival outcomes; which clearly confirm the high interest to introduce viral EBV DNA load as a prognostic biomarker for NPC management.

## Introduction

Nasopharyngeal carcinoma (NPC) represents 23.8% of all head and neck cancers, making it the second most common head and neck squamous cell carcinomas (HNSCCs) after laryngeal cancers [1]. This cancer has a striking geographical distribution; the highest ethnic pattern of incidence arises from Southern Asia, especially in South China where it ranges between 15 and 50 cases per 100,000 of the population. North African countries, some countries of the Arabic peninsula, the Caribbean and the Eskimo lands in Alaska and Greenland are considered areas of intermediate incidence for NPC, with incidence ranging from 3 to 8 cases per 100,000 inhabitants [2,3]. In Morocco, the incidence of NPC in men and women is 4.2/100.000 and 1.2 /100.000, respectively (cancer registry of Casablanca, 2012). Nasopharyngeal carcinoma has a multifactorial etiology; a close association between NPC and the oncogenic pathogen Epstein-Barr virus (EBV) was largely established. Furthermore, environmental and genetic components were also involved in the development of this malignancy [3-6]. However, whatever the geographic distribution and the risk factors of NPC, management of NPC presents a real challenge. Nasopharyngeal carcinoma diagnosis is often difficult and late due to the profound location of the nasopharynx and the nonspecific nature of the clinical symptoms, causing treatment failure and high rate of mortality [4]. Globally, and due to its inherent anatomic constraints and high degree of radio-sensitivity, NPC is mainly treated with concurrent chemo-radiotherapy [5]. Unfortunately, the rate of local recurrence and distant metastasis after 5 years of the initial treatment is still high and ranges from 8.2 to 22.0%. Biomarkers for monitoring therapeutic efficacy and recurrence at follow-up time points are therefore essential for the management of NPC [6].

Currently, it´s widely accepted that EBV is the corner stone in NPC initiation, development and progression, and was therefore the central key in the development of various strategies for NPC diagnosis, follow-up and prognosis. Recent advances in molecular biology have made an outstanding contribution to our understanding of genetic and immunological pathways in NPC development highlighting some interesting biomarkers that could be used in the early diagnosis, effective prognosis and/or as therapeutic targets [7]. In this field, growing interest was given to the assessment of plasmatic EBV viral load as a biomarker for screening, diagnosis and monitoring of NPC, and reported results clearly showed that evaluation of EBV DNA loads using the quantitative PCR is highly sensitive and specific as compared to the serological tests [7,8]. Accordingly, it has been reported that high levels of cell-free EBV DNA before chemo-radiotherapy predict a poor prognosis. Moreover, a detectable cell-free EBV DNA at the end of the treatment appears to be a very good indicator of disease recurrence and distant metastasis [6]. During last decades, a great number of studies have been conducted to evaluate the use of EBV DNA load in the monitoring of NPC and consistent results convert to suggest the EBV DNA load quantification as a promising biomarker for NPC diagnosis and prognosis. However, these studies present a great heterogeneity regarding the cutoff used in the evaluation of the prognosis value of EBV DNA load, the protocol used for DNA extraction and EBV DNA quantification approach. The present study was planned to conduct a meta-analysis to evaluate the prognostic value of the EBV DNA load testing and to assess the interest of using this molecular approach in the diagnosis and prognosis of NPC for a better management of this disease.

## Methods

**Search strategy:** the present meta-analysis was performed according to PRISMA guidelines and all studies focusing on EBV DNA quantification in NPC were discerned. Systematic search strategies were conducted via a range of online literature databases prior to May 2020, including PubMed, Embase, Google scholar and Web of Science. Peer-reviewed literature was retrieved based on the following key words in all databases: (“nasopharyngeal carcinoma” or NPC) and (“EBV DNA load” or “Epstein-Barr virus DNA” or “Epstein-Barr virus DNA load”). Additional studies not covered by the adopted search strategy, were further identified by examining the bibliographies of relevant papers. A table reporting all used papers and the databases used to extract them was added as Annex 1.

**Inclusion and exclusion criteria:** in this meta-analysis, all available descriptive and analytic studies satisfying at least one of the following criteria were included: i) Plasma EBV DNA load was quantified; ii) the association between different clinical outcomes of treatment (overall survival (OS), disease free survival (DFS), progression-free survival (PFS), loco-regional relapse free survival (LRFS), distant metastasis-free survival (DMFS) and EBV DNA levels was analyzed; iii) high pre-treatment EBV DNA (pre-EBV DNA)/post-treatment EBV-DNA (post-EBV DNA) versus low pre-EBV DNA/ post-EBV DNA were compared; iv) hazard ratios (HRs) and 95% confidence intervals (CIs) of outcomes were mentioned. In addition, meta-analyses, reviews, comments, conference abstracts, case reports and studies not reporting basic and informative data were excluded.

**Data extraction:** with respect to the inclusion criteria, available data from all included studies were extracted by two independent authors and in case of any inconsistency, a third author was consulted. The following data were retrieved from eligible studies: name of the first author, year of publication, geographical location of the study, study period, study design, number of patients and controls. Additional data regarding published results were also extracted including the median follow-up time, cutoff values of EBV DNA load, pre and post-EBV DNA levels, survival outcomes (OS, DFS, PFS, LRFS, and DMFS), statistical evaluations (HRs and 95% CIs).

**Endpoints definition:** the main endpoint of OS was defined as the time from random assignment until death. While survival patients were censored at the date of the last follow-up. Overall, DFS was defined as the time from randomization until disease recurrence or death from any cause. Progression-free survival corresponded to the time from randomization until tumor progression or death, whichever occurs first. Loco-regional relapse free survival was determined as the time from randomization to first loco-regional recurrence or death, while DMFS was defined as the time from randomization to the first detection of distant metastasis on imaging or death. Of note, DFS and RFS reported in some studies were used to represent DMFS and LRFS or PFS, respectively.

**Statistical analysis:** the present meta-analysis was performed using review manager (RevMan) (version 5.3, the Cochrane Collaboration, Oxford, England). Pooled HRs and corresponding 95% CIs were used to evaluate the association between pre and post-EBV DNA load and survival outcomes. HR>1 indicates higher risk for the occurrence of events (e.g., distant metastasis, death, relapse, progression, etc). Heterogeneity among studies was evaluated using the Q test, and the statistical significance was set to p-value<0.1. The I^2^index was then used to evaluate the proportion of variation within studies. An I^2^ below 50% and p>0.1 indicated an absence of heterogeneity, while an I^2^ranging between 50% and 100% or p<0.1 indicated a presence of heterogeneity. We also conducted a subgroup meta-analysis study to evaluate whether cutoff of pre-EBV DNA load may have affected the pooled results. To this end, studies were divided into two groups, a first group using a cutoff >1500 copies/ml and a second group using a cutoff ≤1500 copies/ml. Another subgroup analysis was carried out based on follow up duration, one group of studies with a follow up less than 3 years and the other one of studies with a follow up of more than 3 years. Furthermore, funnel plots were generated to evaluate small-study bias visually, and Begg´s test was used to examine the potential publication bias statistically. Finally, meta-regression and subgroup meta-analyses were carried out to assess the potential studies sources of heterogeneity and confounding effects.

## Results

**Figure 1:**
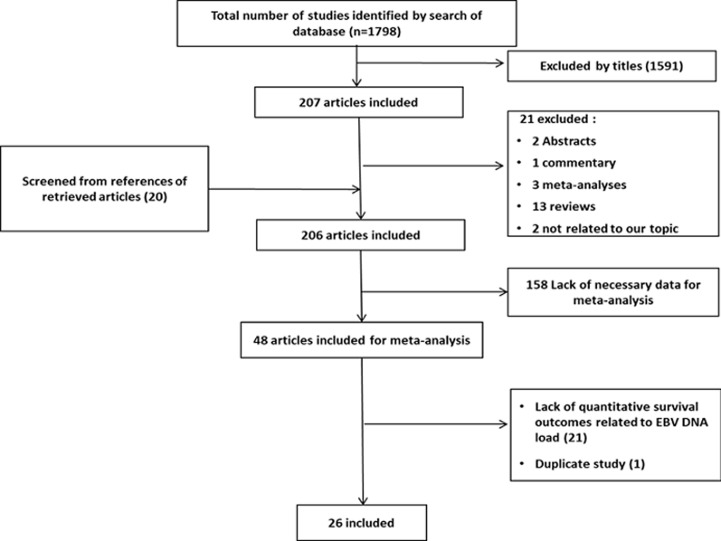
flow diagram of study selection

**Study selection and characteristics:** systematic search strategies conducted in the various online literature databases have led to an initial screening of 1798 studies. After screening of titles and abstracts, 1591 were removed. From the remaining 207 articles, 21 were excluded: 2 papers were limited to the abstracts, 1 commentary, 13 were review papers, 3 meta-analyses and 2 were not related to our topic. Moreover, 206 articles were obtained as initial database after adding 20 studies from references of retrieved articles. Following a detailed evaluation of these 206 studies with respect to adopted inclusion/exclusion criteria, 158 articles lacked necessary data, 21 lacked quantitative survival outcomes related to EBV DNA load and one found to be a duplicate article were excluded. A total of 26 relevant articles studying 9966 patients were included for the final analysis (Figure 1). Among the 26 eligible studies, 16 were retrospective and 10 prospective studies [2,8-32]. Total subjects per study varied between 34 and 1501 patients, with only 2 studies conducted on a sample size exceeding 1000 patients. The characteristics of these studies are reported in Table 1 and show that most studies were conducted in high NPC-endemic areas; 20 in China, 3 in Taiwan and 2 in Thailand. Only 1 study was performed in a low-endemic country and was conducted in patients from Netherlands. The cut-off value of pre-EBV DNA for predicting outcomes varied across studies and ranged between 0 and 10000 copies/ml; the most common employed values being 1500 and 4000 copies/ml. The median follow-up duration ranged from 25 months to 62 months.

**Table 1:** global characteristics of eligible studies selected for the meta-analysis

Authors	Year	Locationn	Study design	Inclusion period	Number of patients	Pre-cutoff	Post-cutoff	Survival outcome	Follow-up (median)
Lin *et al*.	2004	China	Prospective	1999-2002	99	1500	0	OS, PF	30 (14-48)
Chen *et al*.	2016	China	Retrospective	2008-2012	404	4000	-	OS, DMFS,PFS	33.5
Lan *et al*.	2016	China	Retrospective	2007-2011	755	10000	-	DFS, DMFS,	50 (3-87)
Peng *et al*.	2016	China	Retrospective	2009-2012	584	2010	2	OS, DFS, LRFS, DMFS,	38.2 (4.6-58.6)
Jin *et al*.	2017	China	Retrospective	2009-2012	1036	1500	-	OS, DMFS,PFS	60.1 (1.3-79.5)
Stoker *et al*.	2016	Netherland	Prospective	2009-2013	72	2000	-	OS, DFS,	25
Xu *et al*.	2015	China	Retrospective	2006-2011	722	1500	-	OS,LRFS, DMFS,PFS	51.8 (1.7-136.5)
Wen-yi Wang *et al*.	2013	China	Prospective	-	210	1500	-	OS, PFS	-
Xu *et al*.	2014	China	Retrospective	2006-2010	356	1500	-	OS, DMFS,PFS	-
Lv *et al*.	2016	China	Retrospective	2009-2012	1501	4000	-	OS,LRFS, DMFS,PFS	48.4 (1.3-76.4)
Wen-yi Wang *et al*.	2016	China	Retrospective	-	931	100	-	OS	99
Zhao *et al*.	2015	China	Retrospective	2006-3013	637	1500	-	OS, PFS	-
Shen *et al*.	2015	China	Retrospective	2007-2011	89	7500	-	OS, DMFS,PFS	44.9
Wei *et al*.	2014	China	Retrospective	2006-2009	214	1500	-	OS,LRFS,DMFS, PFS	-
Leung *et al*.	2014	China	Prospective	2004-2006	107	4000	-	OS,LRFS,DMFS,PFS	73(4.9-89.6)
Twu *et al*.	2007	Taiwan	Retrospective	-	114	1500	-	OS, PFS	46 (22-67)
Chan *et al*.	2002	China	Prospective	1997-1999	170	4000	500	OS,LRFS,DMFS,PFS	29 (9.25-59.75)
Zhang *et al*.	2016	China	Retrospective	2010-2011	273	-	0	OS, DMFS,DFS	38.4 (5.13-57.4)
Wang *et al*.	2016	Taiwan	Prospective	2007-2016	178	5000	-	OS, DMFS,	57 (25- 117)
Prayongrat *et al*.	2017	Thailand	Retrospective	2010-2015	204	0	-	OS, DMFS,PFS	35.1 (1.6-77.4)
Hsu *et al*.	2011	China	Prospective	2007-2010	73	5000	-	OS	31 (20-40)
Wen-yi Wang *et al*.	2010	Taiwan	Prospective	2005-2008	34	1500	-	OS	30 (18-50)
Huang *et al*.	2019	China	Retrospective	2012-2015	278	7000	0	OS, DMFS,DFS,LRFS	38 (5-67)
Chen *et al*.	2018	China	Prospective	2007-2012	419	1500	-	OS, PFS	56.7
Liu *et al*.	2019	China	Retrospective	2008-2016	401	-	-	OS, DMFS,LRFS	32 (3- 118)
Lertbutsayanukul *et al*.	2018	Thailand	Prospective	2010 - 2015	105	2010	-	OS,DMFS,PFS	45.3

**Pre-EBV DNA levels and survival outcome:** pooled analyses of the 26 selected studies showed that pre-EBV DNA presents a significant prognostic value, with HR (95% CI) of 2.09 [1.74, 2.51] for OS, 1.77 [1.19, 2.62] for DFS, 2.53 [2.18, 2.92] for DMFS, 1.78 [1.45, 2.19] for LRFS and 2.17 [1.91, 2.47] for PFS, indicating that high levels of pre-EBV DNA were significantly associated with higher risk of death, recurrence and distant metastasis in the early phases of NPC management (Table 2). Furthermore, heterogeneity test revealed a significant heterogeneity between-studies in OS, DFS and pre-EBV-DN A with I^2^=48%, p=0.007 and I^2^=65%, p=0.04, respectively; thus, a random effect model was applied to calculate the association of pre-EBV DNA with these parameters. A fixed effect model was used for DMFS, LRFS and PFS as there was no sign of heterogeneity among the studies with I^2^=18%, p=0.26, I^2^=11%, p=0.34 and I^2^=0%, p=0.82 respectively (Table 2).

**Table 2:** association between pre/post-EBV DNA and survival outcomes

Outcomes	Number of studies	Model	HR (CI 95%)	P-value	Heterogeneity tests	
					I^2^ %	P-value
**Pre-EBV DNA**						
OS	22	R	2.09[1.74,2.51]	0.00001	48%	0.007
DFS	4	R	1.77[1.19,2.62]	0.005	65%	0.04
DMFS	15	F	2.53[2.18,2.92]	0.00001	18%	0.26
LRFS	8	F	1.78[1.45,2.19]	0.00001	11%	0.34
PFS	17	F	2.17[1.91,2.47]	0.00001	0%	0.82
**Post-EBV DNA**						
OS	15	R	4.52[2.44,8.36]	0.00001	88%	0.00001
DFS	4	R	4.08[2.38,6.99]	0.00001	72%	0.01
DMFS	9	R	8.88[5.29,4.90]	0.00001	72%	0.0004
LRFS	4	F	1.64[0.99,2.71]	0.05	0%	0.40
PFS	11	R	5.59[3.58,8.71]	0.00001	73%	0.0001

R: random effect mode; F: fixed effect model

**Post-EBV DNA levels and survival outcome:** the association between post-EBV DNA and survival parameters was also assessed. A fixed-effect model was used for LRFS since there was no sign of heterogeneity among the studies (p= 0.40, I^2^=0%). For the other survival outcome parameters, statistical analyses showed significant heterogeneity across the included studies for OS (I^2^=88%, p=0.00001), DFS (I^2^=72%, p=0.01), DMFS (I^2^=72%, p=0.0004) and PFS (I^2^=73%, p=0.0001), and thus random effect model was applied to calculate the association of post-EBV DNA with these parameters. Pooled results clearly showed that high post-EBV DNA load was strongly associated with risk of metastasis with an HR (95%) of 8.88 [5.29,14.90]. In fact, the risk of metastasis was 8-fold higher for patients with high post-EBV DNA levels compared with low post-EBV DNA levels. Post-EBV DNA HR (95% CI) for OS, DFS and PFS were 4.52 [2.44, 8.36], 4.08 [2.38, 6.99] and 5.59 [3.58, 8.71], respectively. However, a weak association between post-EBV DNA and LRFS was obtained with an HR (95%) of 1.64 [0.99 2.71] (Table 2).

**Subgroup analysis based on pre-EBV DNA cutoffs:** to investigate potential sources of heterogeneity within the studies, a subgroup analysis was performed based on EBV DNA load cutoff used in the included studies. Results of the subgroup analyses are reported in (Table 3). Overall, a pre-EBV DNA cutoff ≤1500 copies/ml remains the most used cutoff in the included studies. Results of pre-EBV DNA showed no significant differences in the results of subgroups analysis compared with those of the original analysis. In fact, meta-analysis of pooled results for survival parameters were not affected by the EBV DNA load cutoffs. For the studies using a pre-EBV DNA cutoff of ≤1500 copies/ml, the pooled HR (95% CI) for OS, DMFS, LRFS and PFS were 1.88 [1.56,2.26], 2.39 [1.83,3.11], 1.53 [0.84, 2.76], and 2.07 [1.80, 2.37], respectively. Moreover, for the studies using a pre-EBV DNA cutoff of >1500 copies, the pooled HR and CI for OS, DFS, DMFS, LRFS and PFS were 2.47 [1.84, 3.31], 1.77 [1.19, 2.62], 2.59 [2.17, 3.09], 1.87 [1.41,2.47], and 2.18 [1.78, 2.7], respectively. In addition, no evidence between study heterogeneity was found for pre-EBV DNA ≤1500 associated OS, DMFS and PFS and pre-EBV DNA > 1500 associated DMFS, LRFS and PFS (p<0.01). Although, a very low heterogeneity was found for pre-EBV DNA≤1500 associated LRFS and pre-EBV DNA >1500 associated OS and DFS (p>0.1)

**Table 3:** association between pre-EBV DNA cutoffs and survival outcomes

Outcomes	Number of studies	Model	HR (CI 95%)	P-value	Heterogeneity tests	
					I^2^ %	P-value
**Pre-EBV DNA ≤ 1500**						
OS	10	F	1.88[1.56,2.26]	0.00001	28%	0.18
DMFS	5	F	2.39[1.83,3.11]	0.00001	0%	0.73
LRFS	3	R	1.53[0.84,2.76]	0.16	58%	0.09
PFS	11	F	2.07[1.80,2.37]	0.00001	0%	0.82
**Pre-EBV DNA > 1500**						
OS	11	R	2.47[1.84,3.31]	0.00001	45%	0.05
DFS	4	R	1.77[1.19,2.62]	0.005	65%	0.04
DMFS	10	F	2.59[2.17,3.09]	0.00001	39%	0.10
LRFS	5	F	1.87[1.41,2.47]	0.0001	0%	0.57
PFS	6	F	2.18[1.78,2.67]	0.00001	0%	0.42

R: random effect model; F: fixed effect model

**Subgroup analysis based on follow up duration:** in the present meta-analysis, the included studies were sub-divided in two groups according to the follow up duration; the first subgroup consisted of studies with a follow up less than or equal to 3 years and the second one was studies with a follow up of more than 3 years. Pooled HRs showed that whatever the follow up duration, pre-EBV DNA levels were significantly associated with poorer OS, DFS, DMFS, LRFS and PFS (Table 4). For studies with a follow up duration ≤ to 3 years, our data showed a significant heterogeneity within 14 and 3 studies for OS and DFS parameters, respectively (p>0.01), whereas a very low heterogeneity was found for DMFS, LRFS and PFS parameters (p<0.01). For studies with a follow up duration > to 3 years, no significant heterogeneity within studies was revealed (p<0.01) (Table 4).

**Table 4:** distribution of outcomes parameters according to the follow up duration

Outcomes	Number of studies	Model	HR (CI 95%)	P-value	Heterogeneity tests	
					I^2^ %	P-value
**Follow-up ≤ to 3 years**						
OS	13	R	2.23 [1.61, 3.09]	0.0001	63%	0.001
DFS	3	R	1.97 [1.13, 3.44]	0.02	68%	0.04
DMFS	9	F	2.94 [2.34, 3.70]	0.00001	7%	0.37
LRFS	4	F	2.13 [1.59, 2.37]	0.00001	0%	0.75
PFS	8	F	2.61 [1.99, 3.43]	0.00001	0%	0.90
**Follow-up ≤ to 3 years**						
OS	10	F	2.05 [1.76, 2.37]	0.00001	17%	0.29
DMFS	6	F	2.28 [1.88, 2.75]	0.00001	9%	0.36
LRFS	4	F	1.49 [1.11, 1.99	0.008	20%	0.29
PFS	9	F	2.06 [1.78, 2.38]	0.00001	0%	0.69

R: random effect model; F: fixed effect model

**Figure 2:**
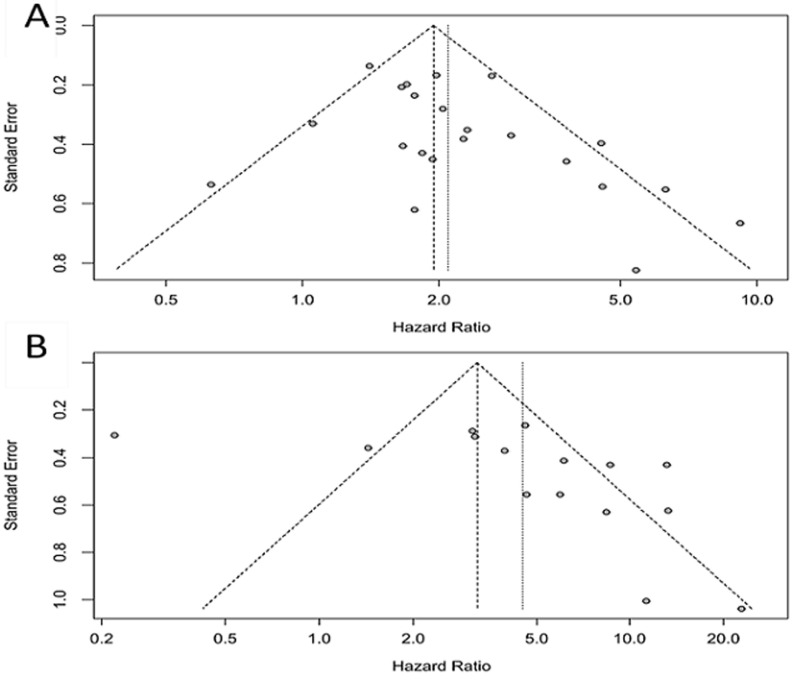
A) the funnel plot of the 22 included studies that reported the association between pre-EBV DNA levels and OS; B) the funnel plot of the 15 included studies that examined post-EBV DNA associated OS

**Assessment of publication bias:** using Begg´s test to analyze publication bias, no evidence of bias was found in pre-EBV DNA associated DFS, DMFS, LRFS (p=0.4634, p=0.5355 and p=0.8593, respectively) and post-EBV DNA associated OS, DFS, DMFS and LRFS (p=0.08663, p=0.4811, p=0.07077 and p=0.4756, respectively) (Table 5). However, a publication bias was observed in the pre-EBV DNA OS (p= 0.03983) and PFS (p=0.006583), and post-EBV DNA PFS (p=0.04864) (Table 5). The funnel plots of the 22 included studies reporting the association between pre-EBV DNA levels and OS and of the 15 included studies that examined post-EBV DNA associated OS are reported in Figure 2.

**Table 5:** evaluation of publication bias

Outcomes	Number of studies	Publication bias (P-value)
**Pre-EBV DNA**		
OS	22	0.03983
DFS	4	0.4634
DMFS	15	0.5355
LRFS	8	0.8593
PFS	17	0.006583
**Post-EBV DNA**		
OS	15	0.08663
DFS	4	0.4811
DMFS	9	0.07077
LRFS	4	0.4756
PFS	11	0.04864

## Discussion

A close association between EBV infection and the NPC was widely reported. Based on this association, several EBV derived molecules are widely used as biomarkers for NPC management. Early studies highlighted EBV serology as an important biomarker for both screening and early detection of NPC in endemic areas [33-35]. However, the main limitation of these serological tests is their poor capacity to assess therapeutic outcomes for NPC patients. Indeed, scientific evidences have shown that the levels of some antibodies remain high even in patients in complete remission [36,37]. Recently, the discovery of cell- free nucleic acids biomarkers for NPC has revolutionized the disease management. Circulating EBV DNA is one of the most non-invasive studied biomarkers of NPC and clinical usefulness for screening, diagnosis and prognosis of NPC patients in endemic areas is widely documented [36,38]. In the present meta-analysis, we evaluated the pre and post-EBV DNA load testing in order to assess this molecular approach in the prognostic management of this disease. Accordingly, the association between pre and post -EBV DNA load and patient´s outcomes (OS, PFS, DMFS, and LRFS) was performed. This meta-analysis included a total of 16 retrospective and 10 prospective studies including 9966 patients. Our results clearly showed that patients with high levels of pre-EBV DNA had higher risk of death, loco-regional recurrence and distant metastasis, compared to patients with low levels of pre-EBV DNA. Indeed, pooled HR values achieved 2.09 (95% CI=1.74, 2.51, p<0.00001) for OS, 1.77 (95% CI=1.19, 2.62, p<0.005) for DFS, 2.53 (95% CI=2.18, 2.92, p<0.00001) for DMFS, 1.78 (95% CI=1.45, 2.19, p<0.00001) for LRFS and 2.17 (95% CI=1.91, 2.47, p<0.00001) for PFS. Similarly, Weng *et al*. have reported that patients with low pre-EBV DNA levels had longer survival rates compared to those with high pre-EBV DNA [38]. Even though most studies included in this meta-analysis were conducted in endemic areas, studies from non-endemic and middle-endemic areas reported a similar prognostic value of pre and post-EBV DNA load. However, these studies were excluded from this meta-analysis due to the lack of HRs values or the EBV DNA load was measured in nasopharyngeal brushings or biopsies. Of particular interest, a study including a cohort of 36 Western patients with stage IIb-IVb nasopharyngeal cancer, showed that pre-EBV DNA levels monitoring can allow the detection of disease recurrence and metastases [39].

Similarly, Alfieri *et al*. have carried out a study on 130 locally-advanced EBER positive NPC Italian patients and revealed that DFS and OS were significantly longer in patients with negative pre-EBV DNA load (p=0.03 and p=0.02, respectively) [40]. In the same population, Alessi et al. showed that EBV DNA load was significantly associated with DFS (p=0.05) [41]. Furthermore, Mazurek *et al*. highlighted the important role of EBV DNA assessment in the diagnostic of NPC patients with T1-T2 tumors in Poland [42]. Another study from Russia recommended the simultaneous use of plasma EBV DNA loads and VCA/IgA antibody levels as diagnostic and monitoring markers for the undifferentiated type of NPC in non-endemic regions [43]. The measurement of EBV DNA load before and after treatment of 22 consecutive Dutch NPC patients revealed that EBV DNA in plasma became undetectable after treatment, and suggest this marker as useful one in a low NPC risk area [37]. Moreover, an American study highlighted that EBV DNA levels was more informative compared to EA serology for distinguishing remission from recurrence NPC disease [37]. In middle endemic areas; it have been reported that EBV DNA load quantification after treatment may be a good predictor of OS and PFS for Tunisian patients with NPC [44]. The persistence of EBV DNA in blood was found to be a good indicator of therapy failure of patients and most studies reported a significant correlation between EBV DNA in plasma after treatment, OS and DMFS [2,41]. Interestingly, in this meta-analysis, pooled HRs revealed that the risk of mortality and metastasis was respectively 8 and 5 fold higher for patients with high post-EBV DNA levels as compared to those with low post-EBV DNA levels. Measuring the change of EBV DNA loads before and after treatment, may be a helpful non-invasive and fast tool to attempts loco-regional and distant recurrences after chemo-radiotherapy.

Difference between these studies (including study design, sample size, ethnicity) has been widely reported and discussed. These differences could be potential sources of heterogeneity between results of studies. In this field, analysis of data from this meta-analysis also indicates a significant heterogeneity between pre and post-EBV DNA loads and most of clinical outcomes (I^2^>50%, p<0.1). To explore the source of this heterogeneity and possible publication bias, we have conducted subgroup analysis based on cutoff values and follow up duration. Our results of subgroups analysis show that pre-EBV DNA ≤1500 or >1500 copies/ml was significantly associated with survival outcomes. Of particular interest, no significant difference in the results of subgroups analysis compared with those of the original analysis was observed. Worldwide, the cutoff for EBV DNA load assessment is well discussed and documented. Although there is no international recommendation regarding the optimal EBV cutoff points for pre-EBV DNA, studies used commonly the cutoff values of 1500 copies/ml and 4000 copies/ml [45]. Accordingly, 10 studies from this meta-analysis used a cutoff of 1500 copies/ml and four used 4000 copies/ml as cutoff for survival analysis. Recently, Lertbutsayanukul *et al*. have analyzed a series of cutoffs (0, 1500, 2010, 2300, 4000 and 50000 copies/ml) and suggested 2300 copies/ml as the optimal value in terms of sensitivity and specificity for predicting 3 years OS, PFS and DMFS [46]. There´s therefore evidence that more studies are needed to establish a standardized cutoff, an optimum pre-EBV DNA cutoff values may serve as guidance for NPC disease risk stratification, combined to TNM staging or alone.

In follow up duration subgroups, significant heterogeneity was found only for the OS and DFS, and no significant heterogeneity within studies with a follow up duration superior to 3 years was observed, suggesting that duration of follow-up couldn´t be a potential source of heterogeneity, and others sources of heterogeneity should be explored. In a previous meta-analysis, the nature of samples (plasma or serum), tumor grade, cutoff values of pre-EBV DNA levels (<1500, ≥1500, <4000, and ≥4000 copies/ml) and detection time of post-EBV DNA levels (1 week, 5 or 8 weeks and 3 months after treatment) were found to be potential sources of heterogeneity [45,47]. Interestingly, another meta-analysis highlighted that detection of EBV DNA in both plasma or serum had higher sensitivity and specificity in prognosis of NPC, but EBV DNA in plasma was found to have a higher accuracy than in serum [47]. On the other hand, Zhang *et al*. have reported that tumor grade, cutoff value and histological differentiation influence moderately, but not significantly, the heterogeneity between studies [45]. Moreover, Qu *et al*. recommended to use any detection time point for post-EBV DNA measurement, whatever 1 week or 3 months after treatment [48]. Overall, we observed that the heterogeneity between studies affect poorly the sensitivity and specificity of this biomarker, and suggest that, whatever the quantification method or the cutoff used, the prognostic utility of EBV DNA load in NPC still highly performing. The present meta-analysis is very informative and highlights the impact of high EBV DNA load in NPC persistence and treatment failure. However, there are still some limitations to be considered for further investigations: i) most of the included subjects were Asiatic patients, neglecting the ethnic and genetic varieties that can influence the expression levels of the EBV DNA loads; ii) patient management, from diagnosis to the end of follow-up, could be largely diverse between the different medical centers, which may influence the pooled results; iii) the association between DFS and pre-EBV DNA ≤1500 wasn´t assessed because of the lack of studies.

## Conclusion

This meta-analysis demonstrated that pre-EBV DNA load can predict patient prognosis at the early step of NPC management. Moreover, post-EBV DNA levels can be an effective tool for detection of recurrence or metastasis in post-treatment surveillance, besides imaging exams (MRI, TEP-CT).

**Funding:** the present research project was funded by Cancer Research Institute (IRC). Ref: 201932.

### What is known about this topic

- Most of the patients with Nasopharyngeal carcinoma have poor prognostic;
- Plasmatic EBV viral load was suggested as a molecular biomarker for NPC diagnosis and monitoring.

### What this study adds

- The present meta-analysis summarized data of a total of 16 retrospective and 10 prospective studies including 9966 patients;
- Patients with high levels of pre-EBV DNA had higher risk of death, loco-regional recurrence and distant metastasis, compared to patients with low levels of pre-EBV DNA;
- The risk of mortality and metastasis was respectively 8 and 5 fold higher for patients with high post-EBV DNA levels as compared to those with low post-EBV DNA levels; high pre-EBV DNA load and detectable post-EBV DNA load is associated with NPC persistence and treatment failure.

## Annex

**Annex 1**: list of papers used in the meta-analysis and the databases used for their extraction (PDF-336kb)
